# Transcriptional expression changes during compensatory plasticity in the terminal ganglion of the adult cricket *Gryllus bimaculatus*

**DOI:** 10.1186/s12864-021-08018-x

**Published:** 2021-10-14

**Authors:** Meera P. Prasad, Donald K. E. Detchou, Felicia Wang, Lisa L. Ledwidge, Sarah E. Kingston, Hadley Wilson Horch

**Affiliations:** 1 Department of Biology, Bowdoin College, 6500 College Station, Brunswick, ME 04011 USA; 2 Present address: School of Marine Sciences and Darling Marine Center, University of Maine, 193 Clarks Cove Rd, Walpole, ME 04573 USA; 3 University of California Santa Cruz, Ecology and Evolutionary Biology Department and UC Natural Reserves, 1156 High St, Santa Cruz, CA 95064 USA

**Keywords:** RNA-seq, Plasticity, Cercal escape system, GO term analysis, Differential expression analysis

## Abstract

**Background:**

Damage to the adult central nervous system often leads to long-term disruptions in function due to the limited capacity for neurological recovery. The central nervous system of the Mediterranean field cricket, *Gryllus bimaculatus*, shows an unusual capacity for compensatory plasticity, most obviously in the auditory system and the cercal escape system. In both systems, unilateral sensory disruption leads the central circuitry to compensate by forming and/or strengthening connections with the contralateral sensory organ. While this compensatory plasticity in the auditory system relies on robust dendritic sprouting and novel synapse formation, the compensatory plasticity in the cercal escape circuitry shows little obvious dendritic sprouting and instead may rely on shifts in excitatory and inhibitory synaptic strength.

**Results:**

In order to better understand what types of molecular pathways might underlie this compensatory shift in the cercal system, we used a multiple k-mer approach to assemble a terminal ganglion transcriptome that included ganglia collected one, three, and 7 days after unilateral cercal ablation in adult, male animals. We performed differential expression analysis using EdgeR and DESeq2 and examined Gene Ontologies to identify candidates potentially involved in this plasticity. Enriched GO terms included those related to the ubiquitin-proteosome protein degradation system, chromatin-mediated transcriptional pathways, and the GTPase-related signaling system.

**Conclusion:**

Further exploration of these GO terms will provide a clearer picture of the processes involved in compensatory recovery of the cercal escape system in the cricket and can be compared and contrasted with the distinct pathways that have been identified upon deafferentation of the auditory system in this same animal.

**Supplementary Information:**

The online version contains supplementary material available at 10.1186/s12864-021-08018-x.

## Background

Damage to mature nervous systems typically leads to profound functional loss from which recovery is difficult [1, 2]. The Mediterranean field cricket *Gryllus bimaculatus*, possesses an unusual level of plasticity after sensory system damage as can be seen in both the adult auditory system [3–6] and the adult cercal escape system [7, 8]. The adult cricket auditory system is capable of compensating for the unilateral loss of an ear with robust dendritic sprouting of deafferented dendrites followed by de novo synapse formation with the contralateral afferents [3, 6]. Crickets also show compensatory plasticity in escape responses after unilateral removal of one of the wind-sensitive appendages known as a cercus, though this compensation relies on synaptic strength alterations [9] instead of obvious anatomical reorganization. Comparing and contrasting the two different compensatory strategies in use in these sensory systems will provide insights into the various mechanisms nervous systems can employ to recover from damage.

Wind-evoked escape responses in many insects are governed by the cercal system, a low-frequency, near-field extension of the animal’s auditory system comprised of cerci, two antenna-like appendages located on the posterior end of the abdomen, that serve as receptor organs [10, 11]. Each cercus has hundreds of mechanoreceptor hairs that detect and integrate directional information from air currents produced by predators and trigger an appropriate escape response [10, 11]. These hairs are innervated by sensory neurons which relay wind direction information to the terminal abdominal ganglion (TAG) forming a map of direction sensitivity [12]. These afferents synapse with approximately 30 local interneurons and eight pairs of ascending giant interneurons (GIs), which project to the thorax and brain [10]. Though the exact nature of the GI circuitry isn’t fully understood, several of these GIs have been shown to respond most strongly to activation of the ipsilateral cercus, with only small responses induced by activation of hairs on the contralateral cercus [8, 13, 14]. If the ipsilateral cercus is removed, however, GIs receive stronger than normal excitatory signals from the remaining contralateral cercus [7–9]. In animals ablated as juveniles, this physiological shift translates into recovered rates of behavioral responses after 6 days and recovered oriented responses after 14 days [15].

Unlike the plasticity described in the auditory system, the compensatory response of the escape behavior circuitry does not depend on anatomical sprouting. Instead, physiological evidence indicates that decreases in inhibition unmask contralateral wind-sensitive inputs [9]. A variety of theories have been proposed to explain the mechanisms responsible for the observed deafferentation-induced loss of inhibition. For example, there might be post-synaptic alterations, such as a reduction in dendritic diameter, which would reduce the effectiveness of inhibitory synapses in these locations. Alternatively, alterations involving a reduction in inhibition supplied by the polysynaptic inhibitory pathways could release the interneurons from inhibition [9]. Given the polysynaptic nature of the presynaptic inhibition, these alterations could be located in the afferent to inhibitory neuron synapse, or in the inhibitory synapses made onto the interneurons themselves.

In an attempt to identify the molecular changes that might be involved in this plasticity, we used the software package Trinity to complete a de novo assembly of a TAG transcriptome that included ganglia from adult male crickets collected one, three, and 7 days after removal of a single cercus. Control tissue was TAGs from animals in which the hair-free, distal tip of the cercus was cut one, three, and 7 days before collection. We used EdgeR and DESeq2 to complete a differential expression analysis and examined Gene Ontology (GO) terms to identify potential molecular pathways that might be involved in this plasticity. Specifically, we screened our differential results for candidates that might be expected to influence synaptic strength or the balance between excitation and inhibition. Our GO term analysis indicates that many members of the ubiquitin proteosome system (UPS), known to play a role in synaptic remodeling [16], and components of the chromatin-mediated transcriptional pathways were differentially expressed after deafferentation. Surprisingly, we see evidence for the differential regulation of GTPase-related factors as well. We discuss the potential role of these identified transcripts in the compensatory plasticity of the cercal escape pathway in the cricket.

## Results and discussion

**Fig. 1:**
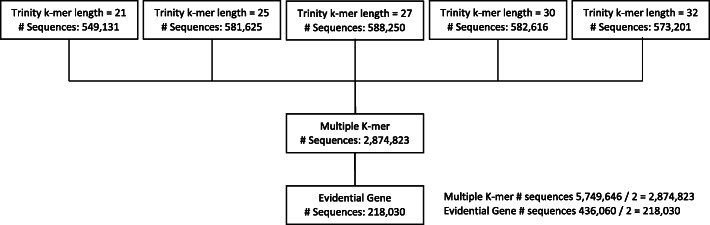
Flow-chart detailing multiple k-mer assembly. Trinity was used to assemble five individual transcriptomes each ato different k-mer lengths. All transcriptomes were combined and then subjected to the Evidential Gene tr2aacdsmRNA classifier to produce one, non-redundant assembly consisting of 218,030 sequences

We performed next generation RNA sequencing on individual terminal ganglia (TAG) from *Gryllus bimaculatus* in order to assess the differential expression of genes after unilateral cercal removal. Thirty samples, each consisting of the TAG from a single adult male cricket in a control or deafferented condition, were used as the source of RNA for transcriptome assembly. Reads were collectively assembled using Trinity, a total of 838,991,089 trimmed and quality filtered paired-end reads of 150 bp in length were input into Trinity for de novo assembly. We built five de novo transcriptomes using five different k-mer lengths: 21, 25, 27, 30, and 32 (Fig. 1). Multiple k-mer methods are shown to be advantageous to a single short or long k-mer assembly alone, improving the diversity and contiguity of the transcripts [17]. We combined all five assemblies into a single reference transcriptome and used the EvidentialGene *tr2aacds.pl* mRNA classifier to reduce redundancies and fragments [18]. This approach is comparable to one used for the re-assembly of the *Gryllus bimaculatus* prothoracic ganglion transcriptome (Wang et al., *submitted*) and will enable comparisons between these two transcriptomes in future studies.

Individual assemblies had an N50 ranging from 1072 to 3027, and as k-mer size increased, the N50 generally increased as well (Table 1). The median, average, and maximum contig length also increased with increasing k-mer length. The total number of Trinity “genes” ranged from 413,346 to 471,672, with higher k-mer assemblies resulting in fewer predicted genes. The GC content remained consistent across each assembly at around 38%. The overall alignment was around 97–98.5% with multi-mapping percentages ranging from 63 to 83% (Table 1). The Trinity assembly approach uses a conservative process to identify unique transcripts, resulting in high redundancies within each assembly [19].

**Table 1:** Multiple k-mer assembly statistics

	K-mer = 21	K-mer = 25	K-mer = 27	K-mer = 30	K-mer = 32
**Total # bases assembled**	389,450,068	578,099,292	608,828,249	631,946,102	637,678,571
**Total # assembled contigs**	549,131	581,625	588,250	582,616	573,201
**Total # Trinity ‘genes’**	471,672	427,875	424,223	418,137	413,346
**Total # Trinity transcripts**	68,117	47,537	36,867	35,325	30,180
**Average contig length (bp)**	709.21	993.94	1034.98	1084.67	1112.49
**Median contig length (bp)**	391	413	407	404	407
**Maximum contig length (bp)**	25,218	42,708	27,081	26,868	26,979
**N50 (bp)**	1072	2319	2584	2890	3027
**GC count for complete assembly (%)**	38.94	38.47	38.49	38.51	38.39
**Overall alignment (%)**	97.0	98.39	98.34	98.39	98.36
**Reads mapped 1 time (%)**	28.26	11.42	10.55	9.24	9.51
**Reads mapped > 1 time (%)**	63.30	81.10	82.02	83.48	83.07

Summary metrics for five different de novo transcriptomes built with varying k-mer lengths.

We combined the five transcriptomes to generate a single transcriptome with a total of 2,874,823 contigs (Fig. 1). EvidentialGene produced a main ‘okay’ set, containing 76,448 contigs, and an alternative ‘okalt’ set, containing 141,582 contigs, which were combined to produce a final transcriptome of total 218,030 contigs. The number of contigs in the final transcriptome was 7.58% that of the original number of contigs. The number of Trinity predicted genes after running EvidentialGene was reduced to 126,966 (Fig. 1).

### Differential expression during compensatory plasticity

**Fig. 2:**
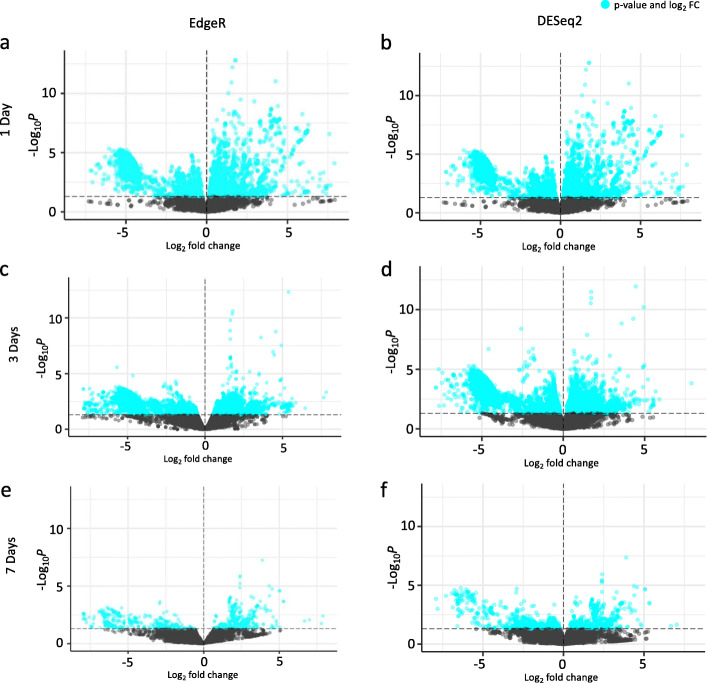
Volcano plots of differential gene expression in control and deafferented terminal ganglia at one, three, or seven days after deafferentation. The horizontal dotted line marks a *p*-value of 0.05, and the vertical dotted line indicates no predicted fold change. Each point represents a predicted transcript determined to be differentially regulated by EdgeR (**a**, **c**, and **e**) or DESeq2 (**b**, **d**, and **f**). Blue points represent predicted transcripts determined to be significantly up- or down-regulated

To determine transcripts that were differentially regulated at one, three, and seven days after deafferentation, the reads for each of the 30 Illumina libraries, excluding three outliers, were mapped back to our multiple k-mer transcriptome to create a counts matrix (See Supplemental Material). Outliers were determined by visualizing MDS plots of the counts data. Three samples (3D_3, 7D_2, and 7D_3) were visually distinct from the rest of the data and were removed (data not shown). Pairwise comparisons of normalized counts data from deafferented vs. control crickets were performed at each time point using both EdgeR and DESeq2 (See Supplemental Materials). We visualized the distribution of upregulated versus downregulated transcripts between the two programs in volcano plots, which indicate that both programs identified a roughly similar proportion of detected transcripts as differentially regulated across all three time points (Fig. 2).

**Fig. 3:**
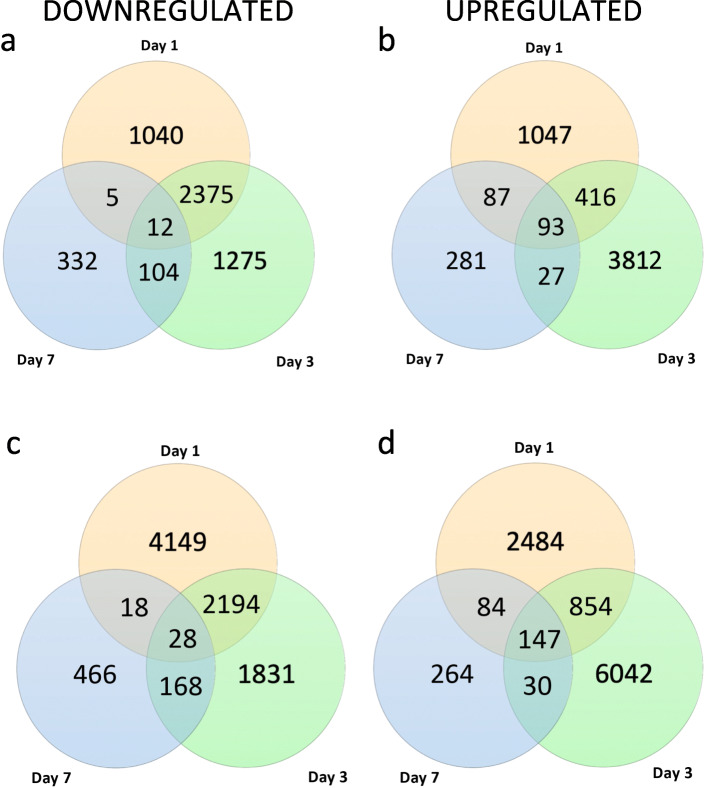
Comparison of differentially regulated genes across three timepoints using EdgeR and DESeq2. Similar patterns in relative numbers of differentially regulated genes were observed between the two programs. **a** EdgeR-identified downregulated genes. **b** EdgeR-identified upregulated genes. **c** DESeq2-identified downregulated genes. **d** DESeq2-identified upregulated genes

**Fig. 4:**
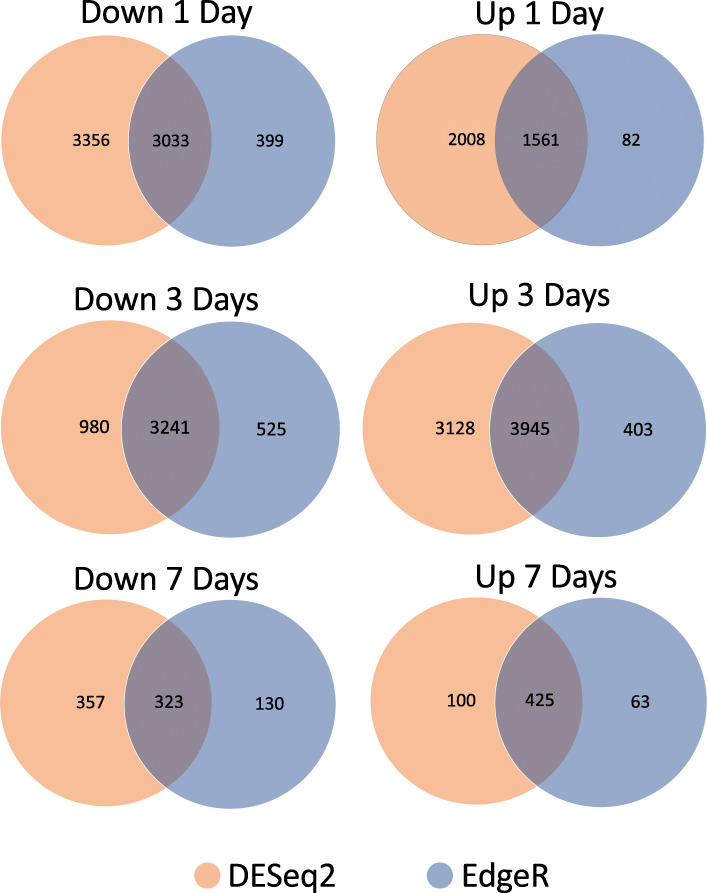
Comparison of the number of differentially regulated genes at one, three, and seven days after deafferentation identified by the two analytical programs, DESeq2 and EdgeR. The number of transcripts found to be differentially regulated by both programs varied by condition, but similar trends were observed across time points. Those transcripts identified by both programs were used for further analyses

Although the two programs generated varying numbers of differentially regulated transcripts, similar patterns in relative numbers across time points were observed (Fig. 3). Both programs showed the largest decrease in transcripts at one and three days, and far fewer at seven days. For the upregulated transcripts (Fig. 3b,d), both programs identified more transcripts upregulated at three days as compared to one and seven days. In both programs, the extent of changes, both upregulated and downregulated, was lowest at seven days. In addition, we compared the transcripts between the two methods at each time point (Fig. 4). For half the time points, the majority of transcripts were identified by both programs; in the other half, more candidates were identified by DESeq2 alone as compared to EdgeR. Regardless, we chose to move forward with those transcripts identified as differentially regulated by both programs. This provided a more limited and conservative transcripts list for further analysis.

### BLAST and gene-ontology analysis

**Fig. 5:**
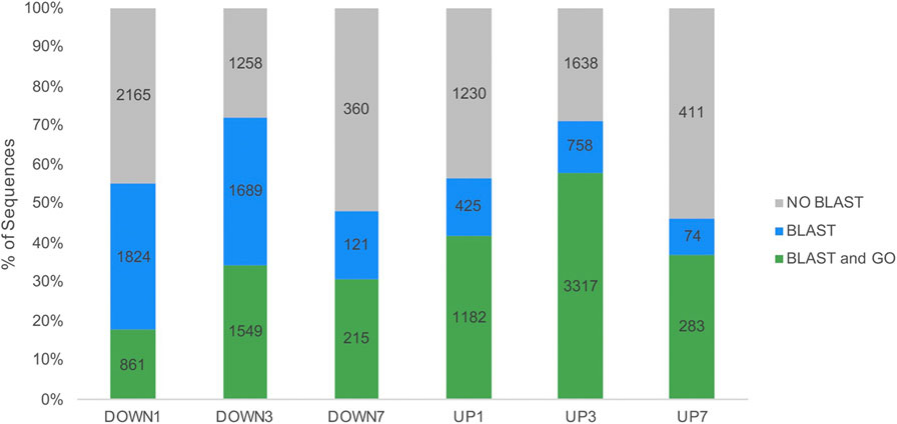
Distribution of transcripts with and without BLAST hits and GO terms. Percent and number of sequences with no BLAST hits, BLAST hits, and BLAST hits with GO term annotation and mapping. Distribution of sequences in each category varies across time points

Once we had a conservative set of transcripts predicted to be differentially regulated, we used BLAST2GO [20] to identify them. Transcripts were BLASTed to the “nr” database, though not all transcripts inputted into the BLAST2GO program resulted in BLAST hits and/or GO annotations (Fig. 5 and Supplemental Materials). At one day downregulated, 18% of transcripts had both BLAST and GO results (green in Fig. 5) and an additional 37% had only BLAST hits (blue in Fig. 5). At three days downregulated, 34% of transcripts had both BLAST and GO results and an additional 38% had only BLAST hits. At seven days downregulated, 31% of transcripts had BLAST and GO results, and an additional 17% had only BLAST hits. At one day upregulated, 41% of transcripts had both BLAST and GO results and an additional 16% had only BLAST hits. At three days upregulated, 58% of transcripts had both BLAST and GO results and an additional 13% had only BLAST hits. At seven days upregulated, 36% of transcripts had BLAST and GO results, and an additional 10% had only BLAST hits (Fig. 5).

A sizeable percentage of differentially regulated transcripts did not match anything in the “nr” database (gray in Fig. 5). There are several potential reasons for this result, none of which are mutually exclusive. For example, some of these transcripts may encode legitimate but uncharacterized proteins. Although we performed poly-A selection as part of the RNA-Seq process, some of these transcripts without BLAST hits may represent non-coding RNAs. Finally, some of these transcripts may be a result of assembly error or may simply be too short to find a match. The candidates without functional information were not included in any further analyses.

### GO term distributions and categories of interest

**Fig. 6:**
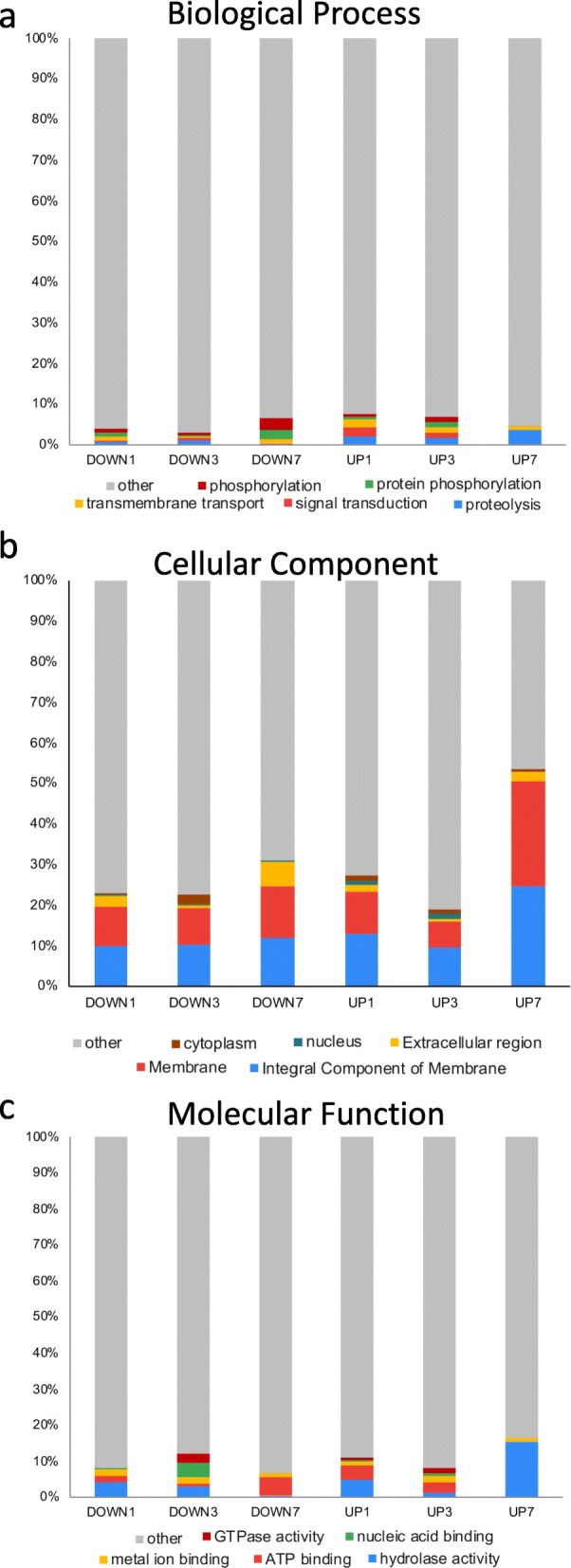
GO term for three root classes of GO terms. The top 5 represented GO terms across all time points related to **a** Biological Process, **b** Cellular Component, and **c** Molecular Function. Many highly represented GO terms were found in the cellular component class whereas a broader range of GO terms were found in the molecular function and biological process classes

BLAST2GO groups GO terms into three root classes: Biological Process (Fig. 6a), Cellular Component (Fig. 6b), and Molecular Function (Fig. 6c). Within these root classes are many subclasses of GO terms. In Fig. 6, we report the five GO terms with the highest number of representatives for each class, though the majority of transcripts are outside these groupings and are indicated as “other” (gray in Fig. 6). Most notably, a large proportion of transcripts fell into either the integral membrane (blue in Fig. 6b) or the membrane component (red in Fig. 6b). In the Molecular Function and Biological Process classes, the top 5 represented GO terms comprised less than 10% of the GO terms identified.

Since compensatory recovery in the cricket escape system is thought to depend on shifts in the balance between excitation and inhibition [9], we searched our GO term lists for functional terms related to synaptic plasticity, such as candidates that might strengthen or weaken synaptic efficacy. We found a number of GO terms related to ubiquitination and the ubiquitin-proteosome system (UPS; Table 2), a system that has been implicated in regulating synaptic strength [16, 21, 22]. Most of the predicted expression changes in ubiquitin-related transcripts in our cricket transcriptome were upregulated 3 days after unilateral removal of a cercus. These results raise the possibility that alterations in the UPS could be responsible for the observed shift in excitatory and inhibitory synapses on GIs after the removal of a cercus [7–9].

**Table 2:** UPS-related GO terms

GO term		DOWN1	UP1	DOWN3	UP3	DOWN7	UP7
GO:0016567	Protein ubiquitination	6	4	1	51	2	0
GO:0006511	Ubiquitin dependent protein catabolic process	0	0	0	47	0	0
GO:0004842	Ubiquitin protein transferase activity	0	0	0	25	2	0
GO:0061630	Ubiquitin protein ligase activity	6	0	0	20	0	0
GO:0005509	Calcium ion binding	37	18	12	105	7	14

Several UPS-related GO terms were identified as differentially regulated at different time points in the terminal ganglion.

The UPS is the main protein degradation system in animal cells, and it has been shown to be a powerful modulator of synaptic transmission in mature synapses. Proteosomes can be recruited and sequestered at local synaptic sites in an activity-dependent manner [23], and the local function of the UPS system impacts synaptic strength and plasticity. For example, altering the level of ubiquitination of proteins at the synapse can alter baseline excitability and synaptic plasticity [24]. These ubiquitin-related changes in synaptic strength can be pre-synaptic or post-synaptic [2]. On the post-synaptic side, glutamate receptor trafficking is known to be regulated by ubiquitination and related enzymes in *C. elegans* [25, 26]. Hypothetically, alterations in the functioning of the UPS in the terminal ganglion GI neurons after cercal removal could weaken inhibitory inputs onto GI interneurons, allowing them to be driven more strongly by the excitatory input from the contralateral side. Future experiments, especially those examining the proteomes of control and deafferented terminal ganglia in the cricket could provide more mechanistic detail regarding which proteins might be regulated by the UPS.

Another large and notable group of transcripts that were differentially expressed were DNA-binding and chromatin-mediated transcription factors (Table 3). Though we don’t yet understand the full function of these transcripts in the cricket nervous system, in other organisms, both alterations in levels of transcription factors [27] and regulation of chromatin [28, 29] can influence the expression of mRNA. Regardless of how mRNA expression is altered, these changes have been shown to influence neuroplasticity [27]. To fully investigate the role of chromatin accessibility, follow-up ATAC-seq experiments would be useful. In addition, we found GO Terms linked to RNA, such as RNA binding (**GO:0003723**), and positive regulation of transcription from RNA polymerase II promoter in response to calcium ion (**GO:006140**) were differentially regulated. Alterations in the expression of RNA-binding factors could lead to shifts in protein translation profiles for a large number of candidates, which could be explored using proteomic tools in the future.

**Table 3:** DNA-binding and chromatin-related GO Terms

GO Term		DOWN1	UP1	DOWN3	UP3	DOWN7	UP7
GO:0003700	DNA-binding transcription factor activity	0	46	1	17	1	0
GO:0003677	DNA-binding	4	48	14	101	3	4
GO:0006355	Regulation of transcription DNA-templated	2	24	10	100	3	0
GO:0032259	Methylation	0	2	7	25	2	0

Several DNA-binding and chromatin-related GO terms were identified as differentially regulated at different time points in the terminal ganglion.

One consistent finding of the studies exploring the compensation in the GI pathways over the past 45 years is the absence of dramatic dendritic sprouting by the giant interneurons [9, 30]. This is notably different from the robust sprouting that has been documented in the prothoracic auditory system after unilateral ear removal in juveniles [31, 32] and adults [3–5]. Thus, when examining the GO terms of our differentially expressed transcripts, we were surprised to see a number of transcripts that might influence morphology. For example, we see predicted changes in terms related to GTPase activity and binding as well as a predicted upregulation of guanyl nucleotide exchange factor (GEF) activity at 3 days (Table 4).

**Table 4:** GTPase-related GO terms

GO term		DOWN1	UP1	DOWN3	UP3	DOWN7	UP7
GO:0005525	GTP binding	11	19	88	105	1	0
GO:0003924	GTPase activity	2	15	84	91	1	0
GO:0005096	GTPase activator activity	0	2	2	17	0	0
GO:0043547	Positive regulation of GTPase activity	0	0	2	45	0	0
GO:0005085	Guanyl nucleotide exchange factor activity	6	0	0	30	0	0

Several GTPase-related GO terms were identified as differentially regulated at different time points in the terminal ganglion.

The Rho family of GTPases have a well-established role in dendritic development and plasticity [33–35]. It is certainly possible that these GTPase-related transcriptional changes are important for deafferentation-induced morphological changes in uncharacterized neurons in this ganglion. It is also possible, however, that these predicted alterations in GTPase levels and activity could result in small and subtle morphological changes in deafferented GIs, which have been described previously. For example, the main dendritic branches of GIs are shorter after deafferentation as compared to controls, and spine-like processes are likely decreased in length after deafferentation as well [9, 30], implying some type of morphological rearrangements. Large scale, contralateral projecting axonal sprouting from the remaining cercus has been reported [14], though the cell bodies for these axons are presumably in the cerci and would not be included in our transcriptome. It is unclear whether these small changes are important for the functional differences observed, but future studies could begin to explore the impact of GTPases on the functional recovery of this system after deafferentation.

It is important to note that the transcriptome and differential expression analyses were performed on the whole terminal ganglia, which could mask important changes that occur in single cells after deafferentation, such as the GIs. Single-cell-RNA-seq analysis of the GIs could help determine whether these changes in expression are occurring for the neurons of interest. However, the weakening of inhibition and the corresponding sensitivity to excitation has also been seen in at least 4 GIs in the TAG upon cercal removal [7, 8]. If the mechanism for the shift in excitation is the same for other GIs in the TAG, it may make it more likely that the expression changes we are detecting in our transcriptome are a result of transcriptional changes in these cells.

### Gene ontology enrichment analysis

Metascape was used to integrate functional enrichment information across independent databases [36] to identify enriched GO terms from our differentially expressed transcript lists. We first reBLASTed our differentially expressed lists against the curated Swiss-Prot database to retrieve appropriate gene identifiers (See Supplemental Materials). Similar ratios of BLAST hit percentages across timepoints were observed using Swiss-Prot as with the “nr” database, however, the percentage of transcripts with Swiss-Prot identifiers was lower than for the “nr” database (Table 5).

**Table 5:** Comparison of transcripts with BLAST results in “nr” versus Swiss-Prot

	DOWN1	UP1	DOWN3	UP3	DOWN7	UP7
**nr (%)**	47.3	51.8	50.4	68.1	38.7	32.6
**Swiss-Prot (%)**	14.9	37.7	16.7	60.2	24.4	24.3

The percentage of transcripts with BLAST results in nr was higher as compared to results obtained in Swiss-Prot across all time points.

**Fig. 7:**
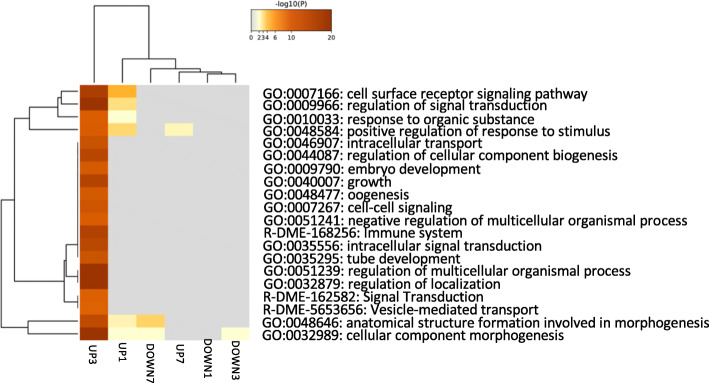
Heatmap of enriched GO terms as determined by Metascape colored by *p*-value. Transcripts identified in *Drosophila melanogaster* were selected and GO-term results correspond to pathways curated in *Drosophila melanogaster*

Despite the low percentage of hits in Swiss-Prot, several categories of GO terms did appear to be enriched, mainly at 3 days (Fig. 7). Most strikingly, a number of GO terms related to developmental processes were enriched, such as embryo development (**GO: 0009790**), growth (**GO: 004007**), tube development (**GO: 0035295**), and cellular component morphogenesis (**GO: 0032989**). These results were a bit surprising, given that this experiment was completed in adult crickets. These results, combined with the GTPase-related factors discussed above, indicate that morphological rearrangements may be associated with the unilateral removal of a single cercus in the adult. Though we do not know if these transcripts are associated with changes in non-GI neurons or potentially influence axonal projections beyond the ganglion, it raises the possibility that developmental signaling mechanisms are recapitulated in the adult in response to the loss of a cercus.

## Conclusion

Unilateral cercal ablation in the cricket, *Gryllus bimaculatus*, leads to a compensatory plasticity response in the escape circuitry of the terminal ganglion. Our transcriptomic analyses identified thousands of transcripts up- and down-regulated after deafferentation. We highlight transcriptional changes related to the proteosome, GTPase signaling, DNA binding, and developmental factors that appeared to be enriched after deafferentation. The data presented here allow the development of targeted hypotheses designed to uncover the mechanisms underlying the deafferentation-induced synaptic plasticity in the terminal ganglion of crickets. The mechanisms at play here can be compared and contrasted with those identified in the prothoracic ganglion of the cricket after unilateral loss of an ear.

## Materials and methods

### Animals and sensory deprivation

Brown-morph Mediterranean field crickets, *Gryllus bimaculatus* (*N* = 30), from an inbred colony, were kept in a twelve-hour light and dark cycle at 50–65% humidity and 26 °C. Adult males were isolated and given cat chow and water ad libitum. Three days after the final adult molt, crickets were cooled and the left-side cercus was removed at the base (“deafferented” experimental condition). In control crickets approximately 0.25 mm was removed from the tip of the left cercus.

### Tissue dissection

Approximately 55 crickets were cooled and immobilized and the terminal ganglia were collected from crickets 1,3, and 7 days after cercal removal (*N* = 5 at each time point) or control amputation (*N* = 5 at each time point). Ganglia were removed by severing the cercal afferents just posterior to the ganglion and then the ventral nerve cord just anterior to the ganglion. Each ganglion was placed into 200 μL QIAzol Lysis Reagent (QIAGEN), homogenized by hand with a pestle, and stored at − 20 °C.

### RNA purification and isolation

RNA was purified from the individual terminal ganglion samples using the QIAGEN RNeasy Lipid Tissue Mini Kit (Qiagen) as specified in the manufacturer’s protocol. Total RNA was purified as specified in the RNeasy protocol, and RNA was eluted from spin columns with 30 μl of RNAse free water. Eluted RNA was treated with TURBO DNAase (Thermofisher). Quality was assessed using an Agilent 2100 Bioanalyzer NANO Chip (Applied Biosystem) and concentration was confirmed using a Qubit 3 fluoruometer (Invitrogen; Table 6). Based on quality and concentration assessments, the best 5 samples per condition (*n* = 30) were selected for sequencing.

**Table 6:** RNA samples

**24-h control**	Cricket 50: 24-h Male, [25 ng/μl]
	Cricket 49: 24-h Male, [21 ng/μl]
	Cricket 51: 24-h Male, [20 ng/μl]
	Cricket 12: 24-h Male, [11 ng/μl]
	Cricket 20: 24-h Male, [10 ng/μl]
**24-h deafferent**	Cricket 40: 24-h Male, [19 ng/μl]
	Cricket 48: 24-h Male, [13 ng/μl]
	Cricket 7: 24-h Male, [12 ng/μl]
	Cricket 41: 24-h Male, [11 ng/μl]
	Cricket 9: 24-h Male, [9 ng/μl]
**3-day control**	Cricket 30: 3-day Male, [18 ng/μl]
	Cricket 17: 3-day Male, [15 ng/μl]
	Cricket 31: 3-day Male, [15 ng/μl]
	Cricket 35: 3-day Male, [14 ng/μl]
	Cricket 32: 3-day Male, [10 ng/μl]
**3-day deafferent**	Cricket 22: 3-day Male, [30 ng/μl]
	Cricket 29: 3-day Male, [12 ng/μl]
	Cricket 19: 3-day Male, [11 ng/μl]
	Cricket 23: 3-day Male, [7 ng/μl]
	Cricket 18: 3-day Male, [6 ng/μl]
**7-day control**	Cricket 25: 7-day Male, [23 ng/μl]
	Cricket 24: 7-day Male, [12 ng/μl]
	Cricket 13: 7-day Male, [11 ng/μl]
	Cricket 36: 7-day Male, [11 ng/μl]
	Cricket 37: 7-day Male, [11 ng/μl]
**7-day deafferent**	Cricket 16: 7-day Male, [22 ng/μl]
	Cricket 28: 7-day Male, [17 ng/μl]
	Cricket 27: 7-day Male, [14 ng/μl]
	Cricket 26: 7-day Male, [13 ng/μl]
	Cricket 11: 7-day Male, [12 ng/μl]

Sample details for prothoracic RNA preparations.

### cDNA library preparation and Illumina sequencing

RNA samples were sent to Georgia Genomics (Athens, GA) for library construction and Illumina sequencing. Samples were prepared according to standard Illumina paired-end library protocols prior to sequencing. Briefly, samples were normalized to 200 ng in 50uL volume. mRNA capture beads were used to select for mRNAs, followed by chemical fragmentation, cDNA synthesis with random priming, adapter ligation, and finally library PCR. Sequencing occurred on the Illumina NextSeq 550 platform running v2 chemistry on a PE150 High output flowcell v2.5 to generate to ~ 35 M paired end reads of 150 bp in length for each sample.

### Data processing and de novo transcriptome assembly

All analyses were performed on the Bowdoin College High Performance Cluster (Microway Quadputer system containing four Intel Xeon E5-4620v2 2.6 GHz eight core CPUs for a total of 32 CPUs and 256Gb of DDR3 1600 MHz ECC/Registered memory). Prior to assembly, raw fastq read files from Illumina sequencing were assessed with FASTQC (v0.11.7) software (Babraham Bioinformatics) to determine the quality distribution, kmer frequencies, and adaptor contamination of our sequences. Quality processing of reads was performed with Rcorrector as well as the python script *FilterUncorrectablePEfasta.py* from the Harvard Informatics Transcriptome Assembly Tools (https://github.com/harvardinformatics/TranscriptomeAssemblyTools). To remove adapter sequences and sequences of a quality score of less than phred 5, we created a perl script using the TrimGalore! software with the cutadapt feature [37]. The max k-mer length was set to 36, and the default minimum k-mer length for the TrimGalore! software was 21. Finally, to remove ribosomal contamination, the sequences were mapped to the SSUParc and LSUParc fasta files from the SILVA ribosomal database [38] using the Bowtie2 software. FASTQC was used to flag the overrepresented sequences, which we then removed using a python script (*RemoveFastqcOverrepSequenceReads.py* from Harvard Informatics Transcriptome Assembly Tools). Transcriptomes were assembled at 5 different k-mer lengths: 21, 25, 27, 30, 32. Trinity (Trinity-v2.6.5) software was run, with a minimum contig length of 200, library normalization with maximum read coverage 50, and RF strand specific read orientation, maximum memory, 250GB, and 32 CPUs for each k-mer assembly. All data associated with this project is available on NCBI (BioProject # PRJNA644928).

### Transcriptome analysis

Individual assemblies were analyzed using the *TrinityStats.pl*. Alignment statistics were obtained using Bowtie2 (v 2.3.4.1) to map raw reads against each de novo transcriptome.

A k-mer identity number was added to each contig’s Trinity ID to allow for future referencing once combined. All five assemblies were concatenated and the Evidential Gene program was used to create a single non-redundant assembly. Evidential Gene works on the longest ORFs, as obtained from the *Transdecoder.LongOrfs* function, removes fragments, and uses a BLAST on self to identify highly similar (98%) sequences. The main (okay) and alternative (okalt) sets output from Evidential Gene were combined into a final fasta file and used as the transcriptome for all future analyses. Bam files, sorted bam files, bam index files, and idxstats.txt files were created using samtools [39]. The metajinomics python mapping tools [40] were used to generate a counts matrix listing the number of reads mapped to each Trinity predicted contig in every cricket sample. Samtools was used to extract the sequencing depth at every base position for each contig in each cricket sample. A python script was used to extract the mean and standard deviation of depth for each contig. The program plotly in R was used to plot the depth of each cricket sample and visually compared to determine outliers. An MDS plot of the counts data was also generated and three samples (3D_3, 7D_2, and 7D_3) were visually distinct from the rest of the data and were removed (data not shown).

### Differential expression

Two programs, EdgeR and DESeq, were used to run the differential expression analysis [41, 42]. Similar filtering and normalization parameters were used in both programs to exclude any contigs that did not have at least one count per million in at least two libraries. Pairwise comparisons at each time point were made between control and deafferented cricket samples to generate lists of significantly upregulated and downregulated genes with a *p*-value cutoff of 0.05. We used the EnhancedVolcano package in R to visualize trends in number of differentially expressed genes per program and time point [43]. Lists of overlapping genes between the two programs for each time point were generated from the pairwise comparisons and used for downstream analysis.

### BLASTing

The NCBI BLASTx local tool [44] was used to identify proteins similar to the translated nucleotide query sequences. An E-value cutoff of 1e-3 was used and max_target_seqs was set to 1. For transcripts with multiple hits we picked the result with the lowest E-value. Query sequences were BLASTed against the entire non-redundant database downloaded from the NCBI website on August 2, 2018. BLAST results were filtered by *p*-value to identify transcripts that were differentially regulated at a *p*-value 0.05 by both EdgeR and DESeq2 programs.

### Gene ontology analysis

BLAST2GO was used to provide GO term annotations for differentially regulated transcripts at each of the time points using the parameters: BLASTx-fast against the nr database, number of BLAST hits = 20, E-value of 1.0 e − 3, word size of 6, hsp length cutoff of 33, with default mapping and annotation settings. GO terms associated with various transcripts were manually grouped according to GO subtype (cellular component, biological process, or molecular function) and plotted to view the distribution across time points. Gene Ontology enrichment analysis was performed with the web-based platform Metascape. The differentially expressed transcripts were BLASTed to the Uniprot/Swissprot database downloaded February 2020 to obtain properly formatted gene ID lists accepted by Metascape.

## Supplementary Information

**Additional file 1: Table S1.** Counts matrix for all samples. **Table S2.** Transcripts identified as significantly upregulated at or below *p* = 0.05, 1 day post cercal removal by DESeq2. Column headings are as follows: qaccver (Query accesion.version) saccver (Subject accession.version), qstart (start of alignment in query), qend (end of alignment in query), sstart (start of alignment in subject), send (end of alignment in subject), bitscore, qframe (Query frame), evalue (expect value), sframe (Subject frame), ssciname (Subject Scientific Name), scomname (Subject Common Name), salltitles (All Subject Titles). **Table S3.** Transcripts identified as significantly upregulated at or below *p* = 0.05, 3 days post cercal removal by DESeq2. Column headings are as follows: qaccver (Query accesion.version) saccver (Subject accession.version), qstart (start of alignment in query), qend (end of alignment in query), sstart (start of alignment in subject), send (end of alignment in subject), bitscore, qframe (Query frame), evalue (expect value), sframe (Subject frame), ssciname (Subject Scientific Name), scomname (Subject Common Name), salltitles (All Subject Titles). **Table S4.** Transcripts identified as significantly upregulated at or below *p* = 0.05, 7 days post cercal removal by DESeq2. Column headings are as follows: qaccver (Query accesion.version) saccver (Subject accession.version), qstart (start of alignment in query), qend (end of alignment in query), sstart (start of alignment in subject), send (end of alignment in subject), bitscore, qframe (Query frame), evalue (expect value), sframe (Subject frame), ssciname (Subject Scientific Name), scomname (Subject Common Name), salltitles (All Subject Titles). **Table S5.** Transcripts identified as significantly downregulated at or below *p* = 0.05, 1 day post cercal removal by DESeq2. Column headings are as follows: qaccver (Query accesion.version) saccver (Subject accession.version), qstart (start of alignment in query), qend (end of alignment in query), sstart (start of alignment in subject), send (end of alignment in subject), bitscore, qframe (Query frame), evalue (expect value), sframe (Subject frame), ssciname (Subject Scientific Name), scomname (Subject Common Name), salltitles (All Subject Titles). **Table S6.** Transcripts identified as significantly downregulated at or below *p* = 0.05, 3 days post cercal removal by DESeq2. Column headings are as follows: qaccver (Query accesion.version) saccver (Subject accession.version), qstart (start of alignment in query), qend (end of alignment in query), sstart (start of alignment in subject), send (end of alignment in subject), bitscore, qframe (Query frame), evalue (expect value), sframe (Subject frame), ssciname (Subject Scientific Name), scomname (Subject Common Name), salltitles (All Subject Titles). **Table S7.** Transcripts identified as significantly downregulated at or below *p* = 0.05, 7 days post cercal removal by DESeq2. Column headings are as follows: qaccver (Query accesion.version) saccver (Subject accession.version), qstart (start of alignment in query), qend (end of alignment in query), sstart (start of alignment in subject), send (end of alignment in subject), bitscore, qframe (Query frame), evalue (expect value), sframe (Subject frame), ssciname (Subject Scientific Name), scomname (Subject Common Name), salltitles (All Subject Titles). **Table S8.** Transcripts identified as significantly upregulated at or below *p* = 0.05, 1 day post cercal removal by EdgeR. Column headings are as follows: qaccver (Query accesion.version) saccver (Subject accession.version), qstart (start of alignment in query), qend (end of alignment in query), sstart (start of alignment in subject), send (end of alignment in subject), bitscore, qframe (Query frame), evalue (expect value), sframe (Subject frame), ssciname (Subject Scientific Name), scomname (Subject Common Name), salltitles (All Subject Titles). **Table S9.** Transcripts identified as significantly upregulated at or below *p* = 0.05, 3 days post cercal removal by EdgeR. Column headings are as follows: qaccver (Query accesion.version) saccver (Subject accession.version), qstart (start of alignment in query), qend (end of alignment in query), sstart (start of alignment in subject), send (end of alignment in subject), bitscore, qframe (Query frame), evalue (expect value), sframe (Subject frame), ssciname (Subject Scientific Name), scomname (Subject Common Name), salltitles (All Subject Titles). **Table S10.** Transcripts identified as significantly upregulated at or below *p* = 0.05, 7 days post cercal removal by EdgeR. Column headings are as follows: qaccver (Query accesion.version) saccver (Subject accession.version), qstart (start of alignment in query), qend (end of alignment in query), sstart (start of alignment in subject), send (end of alignment in subject), bitscore, qframe (Query frame), evalue (expect value), sframe (Subject frame), ssciname (Subject Scientific Name), scomname (Subject Common Name), salltitles (All Subject Titles). **Table S11.** Transcripts identified as significantly downregulated at or below *p* = 0.05, 1 day post cercal removal by EdgeR. Column headings are as follows: qaccver (Query accesion.version) saccver (Subject accession.version), qstart (start of alignment in query), qend (end of alignment in query), sstart (start of alignment in subject), send (end of alignment in subject), bitscore, qframe (Query frame), evalue (expect value), sframe (Subject frame), ssciname (Subject Scientific Name), scomname (Subject Common Name), salltitles (All Subject Titles). **Table S12.** Transcripts identified as significantly downregulated at or below *p* = 0.05, 3 days post cercal removal by EdgeR. Column headings are as follows: qaccver (Query accesion.version) saccver (Subject accession.version), qstart (start of alignment in query), qend (end of alignment in query), sstart (start of alignment in subject), send (end of alignment in subject), bitscore, qframe (Query frame), evalue (expect value), sframe (Subject frame), ssciname (Subject Scientific Name), scomname (Subject Common Name), salltitles (All Subject Titles). **Table S13.** Transcripts identified as significantly downregulated at or below p = 0.05, 7 days post cercal removal by EdgeR. Column headings are as follows: qaccver (Query accesion.version) saccver (Subject accession.version), qstart (start of alignment in query), qend (end of alignment in query), sstart (start of alignment in subject), send (end of alignment in subject), bitscore, qframe (Query frame), evalue (expect value), sframe (Subject frame), ssciname (Subject Scientific Name), scomname (Subject Common Name), salltitles (All Subject Titles). **Table S14.** Blast2GO results for candidates identified as upregulated in the TAG by both EdgeR and DESeq2 1 day after cercal removal. **Table S15.** Blast2GO results for candidates identified as upregulated in the TAG by both EdgeR and DESeq2 3 days after cercal removal. **Table S16.** Blast2GO results for candidates identified as upregulated in the TAG by both EdgeR and DESeq2 7 days after cercal removal. **Table S17.** Blast2GO results for candidates identified as downregulated in the TAG by both EdgeR and DESeq2 1 day after cercal removal. **Table S18.** Blast2GO results for candidates identified as downregulated in the TAG by both EdgeR and DESeq2 3 days after cercal removal. **Table S19.** Blast2GO results for candidates identified as downregulated in the TAG by both EdgeR and DESeq2 7 days after cercal removal. **Table S20.** Swiss-Prot matches for candidates identified as upregulated in the TAG by both EdgeR and DESeq2 1 day after cercal removal. **Table S21.** Swiss-Prot matches for candidates identified as upregulated in the TAG by both EdgeR and DESeq2 3 days after cercal removal. **Table S22.** Swiss-Prot matches for candidates identified as upregulated in the TAG by both EdgeR and DESeq2 7 days after cercal removal. **Table S23.** Swiss-Prot matches for candidates identified as downregulated in the TAG by both EdgeR and DESeq2 1 day after cercal removal. **Table S24.** Swiss-Prot matches for candidates identified as downregulated in the TAG by both EdgeR and DESeq2 3 days after cercal removal. **Table S25.** Swiss-Prot matches for candidates identified as downregulated in the TAG by both EdgeR and DESeq2 7 days after cercal removal.

## Data Availability

The read data described herein is publicly available on NCBI (BioProject # PRJNA644928; https://www.ncbi.nlm.nih.gov/bioproject/?term=PRJNA644928). Assembly data have been uploaded to NCBI and are under embargo until publication.
